# Severe Pain Phenotype in Hemoglobin C (HbC)/Beta-Zero (β⁰) Thalassemia Without Hemoglobin S (HbS): A Clinical and Diagnostic Challenge

**DOI:** 10.7759/cureus.100952

**Published:** 2026-01-06

**Authors:** Michael J Martinez, Benjamin N Friedland, Aayushi Jha, Tanya Zagoruychenko, Sesha Berman

**Affiliations:** 1 Internal Medicine, St. Matthew's University, West Bay, CYM; 2 Internal Medicine, Touro College of Osteopathic Medicine, New York, USA; 3 Internal Medicine, State University of New York (SUNY) Downstate Medical School, Brooklyn, USA; 4 Internal Medicine, Brookdale University Hospital Medical Center, Brooklyn, USA

**Keywords:** compound heterozygosity, extramedullary hematopoiesis, hemoglobin c, transfusion-dependent anemia, β⁰-thalassemia, β-globin gene mutations

## Abstract

Abnormalities in the synthesis or structure of globin chains are known as hemoglobinopathies, and they produce a wide range of clinical phenotypes. When both alleles of the same gene carry different hemoglobin variants, this results in compound heterozygosity. The condition described in this case report (hemoglobin C (HbC)/beta-zero (β⁰)-thalassemia) is one such example. On their own, neither HbC nor β⁰-thalassemia is typically associated with acute vaso-occlusive crises, and the combination of HbC/β⁰-thalassemia has not been well characterized. Limited reports indicate that this genotype is not known to cause pain episodes resembling those seen in HbSC disease. Here, we describe a 32-year-old woman with DNA-confirmed HbC/β⁰-thalassemia. She has chronic transfusion dependence, marrow expansion, and anemia, with a baseline hemoglobin of about 8 g/dL. Despite treatment with hydroxyurea and opioid therapy, she has persistent diffuse bone pain. During the current admission, she presented with worsening pain, nausea, and vomiting. Laboratory findings showed stable hemoglobin compared to her baseline, without leukocytosis or sickling, and imaging revealed no acute pathology. Her pain was managed with intravenous hydromorphone, with plans to transition to oral methadone and oxycodone as needed before discharge. This case illustrates the difficulty of managing severe pain in HbC/β⁰-thalassemia, particularly when symptoms occur with high intensity but without evidence of vaso-occlusion or acute hemolysis. While extramedullary hematopoiesis is a leading explanation for her pain, the degree of pain she experienced is unusual in the absence of acute sickling. Given the rarity of this genotype and the limited amount of published data, management remains challenging. Further research is needed to clarify the mechanisms of pain in HbC/β⁰-thalassemia and to distinguish organic causes from central sensitization in patients with chronic opioid use.

## Introduction

Hemoglobinopathies are a group of inherited disorders caused by qualitative or quantitative abnormalities of globin chain synthesis. They include structural hemoglobin variants, such as hemoglobin S (HbS) and C (HbC), and thalassemias, in which mutations reduce or eliminate the production of either α- or β-globin chains. These disorders can produce a wide spectrum of clinical phenotypes, ranging from asymptomatic states to transfusion-dependent anemias and vaso-occlusive pain crises.

HbC results from a point mutation in codon 6 of the β-globin gene, where lysine is substituted for glutamic acid. This alters red cell physiology by decreasing cell deformability and increasing the tendency to form hexagonal intracellular crystals [1]. However, unlike HbS found in sickle cell disease (SCD), HbC alone does not polymerize with hypoxia and thus typically does not cause classic sickling-mediated vaso-occlusion pain crises or affect life span. Clinically, homozygous HbCC disease can produce mild to moderate hemolytic anemia and splenomegaly, whereas heterozygous HbAC is generally asymptomatic [2].

β-Thalassemia denotes mutations that reduce (β⁺) or completely halt (β⁰) β-globin synthesis. Patients can remain asymptomatic or have severe, transfusion-dependent anemia, hepatosplenomegaly, and marrow expansion, depending on the mutation type. While beta-thalassemias do not cause the vaso-occlusive crises seen in sickle cell patients, they can be associated with musculoskeletal pain related to marrow expansion, skeletal remodeling, and osteoporosis, particularly in transfusion-dependent phenotypes [3]. Consequently, HbC/β⁰-thalassemia is clinically regarded as a benign condition compared to SCD or HbSC disease, though the pain phenotype is poorly defined.

When different structural globin variants are co-inherited, they result in compound heterozygosity. For example, compound heterozygosity for HbSC is a well-recognized sickling disorder in which the vaso-occlusive phenomena occur with appreciable frequency [4]. While HbSC is common and extensively characterized, the combination of HbC/β⁰-thalassemia is not well-studied. The limited data available suggest that HbC/β⁰-thalassemia generally does not lead to pain crises, with only a handful of rare case reports describing such symptoms, including one documented case from London, United Kingdom [5]. Here, we present the case of a 32-year-old woman with HbC/β⁰-thalassemia who presented with severe pain. Through this report, we aim to contribute to the limited literature on symptomatic manifestations of HbC/β⁰-thalassemia occurring in the absence of HbS.

## Case presentation

A 32-year-old woman with DNA-confirmed HbC/β⁰-thalassemia received continuous medical care for her ongoing hemolysis and pain control. The β-globin sequencing results from admission day 1 showed that the patient had two different mutations: c.19G>A (HbC, p.Glu7Lys) and c.118C>T (p.Gln40*, codon-39 stop). The α-globin mutation and deletion/duplication testing results were negative. Serial hemoglobin electrophoreses, most recently on admission day 2, showed HbC 89%-92%, HbA₂ 5%-6%, HbF 2%-6%, and confirmed HbS 0% (Table 1).

**Table 1:** Hemoglobin electrophoresis profile over time (percent of total hemoglobin).

Component	09/10/2025	12/07/2024	Reference range
HbF	2.1	5.6	<1.0%
HbA	0	0	95%-98%
HbA₂	5.7	5.3	2%-3%
HbS	0	0	0%
HbC	92.2	89.1	0%
HbE	0	0	0%
Hb variant	0	0	-

**Figure 1:**
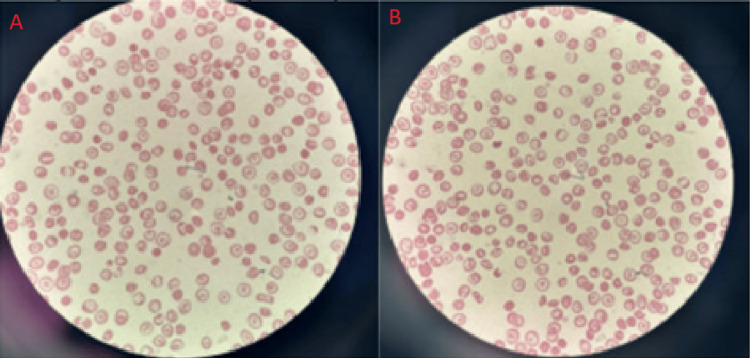
Peripheral blood smear (Wright-Giemsa stain, 100x magnification). Findings include microcytic, hypochromic red blood cells, numerous target cells, and characteristic rectangular hemoglobin C crystals (A, B). Platelets are adequate.

A peripheral smear obtained on admission day 4 showed microcytosis and hypochromia with multiple target cells, elliptocytes, and rectangular HbC crystals; however, platelet counts remained normal (Figure 1).

Baseline laboratory testing showed a hemoglobin level of 8 g/dL, mean corpuscular volume of 54-55 fL, red cell distribution width of 18%-19%, and platelet counts between 120 and 200 × 10³/µL. Abdominal CT performed on admission day 5 demonstrated hepatosplenomegaly (liver 21 cm, spleen 17.5 cm), findings consistent with marrow expansion and extramedullary hematopoiesis, although no discrete paraspinal masses were seen. The original imaging could not be retrieved because it was performed at an external facility that does not grant image access.

The patient has received more than 30 lifetime transfusions and typically requires transfusion every two to three weeks to maintain her hemoglobin above 11 g/dL for functional improvement. She has never received iron chelation therapy and has no documented iron overload, though ferritin levels and T2 MRI were not evaluated during this admission, making it difficult to assess her current iron burden.

She takes hydroxyurea 1,500 mg daily for fetal hemoglobin induction. She reports recurring pain episodes when she stops hydroxyurea, and notes worsening pain when her hemoglobin falls. She has a history of hemolytic crises triggered by stress, weather changes, dehydration, or illness. Prior notes describe icteric sclera, dark urine, extreme fatigue, and hip and leg pain. She previously had high exercise tolerance as an athlete, but now experiences significant limitation due to anemia and chronic pain.

Her ongoing pain typically begins in the lower back and hips and radiates down both legs. Her hematology team attributes this to painful marrow expansion and extramedullary hematopoiesis. Her social situation, including living in a shelter with her mother and four children, working per diem in emergency medical services, and being managed under an opioid behavioral contract, adds complexity when distinguishing organic pain from centralized pain syndromes.

She has had four pregnancies, each requiring third-trimester admission for hemolytic anemia; all deliveries were vaginal. Her past medical history includes asthma and anemia. Surgical history includes laparoscopic endometriosis surgery and fallopian tube reconstruction, with multiple midline catheter placements. She has documented allergies to haloperidol (angioedema), penicillins, metoclopramide, ondansetron, seafood, peanuts (anaphylaxis), and hydroxyzine (hives). Family history is notable for cousins with SCD, though her sister is unaffected.

On admission day 6, she developed worsening diffuse bone pain beyond her typical baseline, unresponsive to home oxycodone and methadone. She also experienced nausea and vomiting that impaired oral intake. She denied fever, respiratory symptoms, chest pain, or leg swelling. Examination showed pain-related distress but no fever, normal oxygen saturation, clear lungs, a soft abdomen with left upper-quadrant fullness, and no neurologic deficits.

Laboratory results from admission days 7-13 showed hemoglobin levels between 8.1 and 9.3 g/dL, hematocrit 24%-27%, and red blood cell counts of 4.39-5.01 × 10⁶/µL. Mean corpuscular volume remained at 54-55 fL, mean corpuscular hemoglobin at ~18 pg, and red cell distribution width at 18%-19%. White blood cell counts ranged from 7.4 to 9.2 × 10³/µL, and platelets from 143 to 190 × 10³/µL. The hemolysis panel showed normal lactate dehydrogenase (173 U/L), bilirubin of 1.4 mg/dL, and a reticulocyte count of 2.67%. Vitamin B12 was low-normal at 282 pg/mL, prompting testing for methylmalonic acid, celiac disease, and intrinsic factor antibodies (Table 2). Imaging included a chest radiograph without acute chest syndrome and hip/pelvis radiographs without evidence of fracture or avascular necrosis. Importantly, these findings were consistent with her baseline and showed no signs of acute hyper-hemolysis or worsening anemia despite her severe reported pain.

**Table 2:** Baseline and admission laboratory results. MCV: mean corpuscular volume, MCH: mean corpuscular hemoglobin, RDW: red cell distribution width, LDH: lactate dehydrogenase.

Parameter	Result	Reference range (units)
Hemoglobin	8.3	12-15.5 g/dL
Hematocrit	25	36%-46%
RBC count	4.5	4.2-5.4 × 10^6^/µL
MCV	54	80-100 fL
MCH	18	27-33 pg
RDW	19	11%-15%
WBC	8.4	4.0-10.5 × 10^3^/µL
Platelets	160	150-400 × 10^3^/µL
LDH	173	100-190 U/L
Total bilirubin	1.4	0.3-1.2 mg/dL
Reticulocyte count	2.7	0.5%-2.5%
Vitamin B12	282	200-900 pg/mL

The patient received intravenous hydromorphone for pain management during hospitalization because her home regimen of methadone and oxycodone was no longer providing adequate relief. She is currently receiving medication every three hours, with a plan to transition to oral methadone 20 mg three times daily, along with oxycodone as needed, prior to discharge. Supportive care includes intravenous hydration, antiemetics (dexamethasone), a bowel regimen, incentive spirometry to prevent atelectasis, and enoxaparin for venous thromboembolism prophylaxis. Management of patients with hematologic conditions also requires regular monitoring with CBC, reticulocyte counts, lactate dehydrogenase, and bilirubin, as well as folate supplementation and hydroxyurea maintenance at safe and appropriate doses. Transfusion was not required during this admission, as her hemoglobin remained close to baseline, vital signs were stable, and her pain gradually improved. She will be considered for discharge once her pain is consistently managed with oral medications and her oral intake is adequate. After discharge, her previously established outpatient transfusion schedule can resume, along with monitoring for iron overload.

## Discussion

This case highlights the rare presentation of a severe pain phenotype in a patient with documented HbC/β⁰-thalassemia, in the absence of acute vaso-occlusion. Notably, this patient demonstrated 0% HbS on hemoglobin electrophoresis. She was admitted for a complaint of diffuse bone pain that was uncontrolled on a reported baseline opioid dose of methadone 20 mg q8h and oxycodone 10 mg q6h prn for breakthrough pain. The patient had several prior admissions, which required IV hydromorphone for pain control, and outpatient pain medications and transfusions are managed by Hematology-Oncology in coordination with a methadone clinic. The absence of documented HbS in conjunction with the lack of clinical signs of vaso-occlusive crisis complicated this case. It prompted further investigation, as the presentation of combination hemoglobin C/β⁰-thalassemia is not well-documented in the literature.

The overlap in symptomology between this case and common presentations of vaso-occlusive crisis secondary to SCD is difficult to explain, given both the lack of documentation of this specific combined disorder and prior knowledge that both β⁰-thalassemia and HbC are not typically associated with acute crises [2,3]. Both conditions are well-documented in isolated cases, as are their associated clinical manifestations. The interaction between HbC and β-thalassemia is poorly understood, but current literature suggests it is a clinically benign condition compared to HbSC disease. In this patient and in the literature, β⁰-thalassemia is known to cause extramedullary hematopoiesis (EMH), which can cause pain and is a leading hypothesis for the source of pain throughout this particular patient’s clinical course [6]. However, confirmatory imaging for EMH (such as MRI showing paraspinal masses) was not available in this admission, limiting our ability to definitively attribute the pain to this mechanism.

Given the discordance between the patient’s severe subjective pain and the lack of objective inflammatory markers (leukocytosis, significant reticulocytosis) or sickling, the differential diagnosis is broad. The patient has a history of chronic high-dose opioid use and is managed with a behavioral contract, suggesting that her pain perception may be heightened independent of acute nociceptive stimuli (central sensitization or opioid-induced hyperalgesia). Furthermore, while plain radiography excluded acute fractures and avascular necrosis, other etiologies such as osteomyelitis or subtle ischemic changes could not be fully excluded without an MRI. This case highlights the risk of treating laboratory-negative pain solely with escalating opioids, which may paradoxically worsen symptoms in the setting of OIH.

One limitation of this case is the subjective nature of pain itself, though commonly used objective markers of sickle cell crisis are reticulocytosis, leukocytosis, elevated LDH, and evident sickling on peripheral blood smear [7]. Additionally, iron overload markers (Ferritin, T2 MRI) were not assessed during this acute admission, limiting our ability to evaluate iron toxicity as a contributing factor to her pain. From a clinical standpoint, it was difficult to correlate reported symptoms with classic signs of pain secondary to crisis. During her clinical course, there were moments when pain crisis markers were within normal limits, and the patient was complaining of severe pain. On the other hand, closer to discharge, there was marked reticulocytosis, though the patient’s pain seemed to be under better control. While extramedullary hematopoiesis remains a primary consideration given her history, it remains a diagnosis of exclusion in this admission due to the lack of acute radiographic confirmation. Cases such as this can sometimes be difficult to navigate from a medical management perspective with the growing concern for overuse of opioids; however, the patient remained in persistent distress secondary to poor control of pain as medications were tapered. This case raises concern as to the potential negative pain outcome for patients with the rare combination of hemoglobin C/β⁰-thalassemia disease, and prompts further investigation into the proper clinical management of discordant pain symptoms in this subset of patients.

## Conclusions

This case illustrates the clinical complexity of HbC/β⁰-thalassemia, a rare heterozygous hemoglobinopathy that is not well-characterized in the literature. Despite the absence of HbS and no evidence of sickling on peripheral smear, the patient experienced pain intensity comparable to a vaso-occlusive crisis, without objective laboratory correlates of hemolysis or inflammation. The severity of her symptoms, combined with the lack of standardized treatment protocols for this genotype, complicated medical management. The discordance between her reported pain and stable laboratory findings highlights the potential contribution of non-hematologic factors, including opioid tolerance and central sensitization, in symptom manifestation. Clinicians should recognize that severe pain in rare hemoglobinopathies may not always indicate acute vaso-occlusion, and careful reliance on objective markers is essential to guide appropriate, multimodal pain management strategies.
